# A Study on the Pathological Effects of Trypanorhyncha Cestodes in Dusky Groupers *Epinephelus marginatus* from the Canary Islands

**DOI:** 10.3390/ani11051471

**Published:** 2021-05-20

**Authors:** Carolina de Sales-Ribeiro, Miguel A. Rivero, Antonio Fernández, Natalia García-Álvarez, Jorge Francisco González, Oscar Quesada-Canales, María José Caballero

**Affiliations:** 1 Veterinary Histology and Pathology, Institute for Animal Health and Food Safety (IUSA), Veterinary School, Universidad Las Palmas de Gran Canaria, 35413 Arucas, Spain; carolina.sales101@alu.ulpgc.es (C.d.S.-R.); antonio.fernandez@ulpgc.es (A.F.); oscar.quesada@ulpgc.es (O.Q.-C.); mariajose.caballero@ulpgc.es (M.J.C.); 2 Division of Infectious Diseases and Ichthiopathology, Institute for Animal Health and Food Safety (IUSA), Veterinary School, Universidad Las Palmas de Gran Canaria, 35413 Arucas, Spain; natalia.garcia@ulpgc.es; 3 Division of Animal Production and Biotechnology, Institute for Animal Health and Food Safety (IUSA), Veterinary School, Universidad Las Palmas de Gran Canaria, 35413 Arucas, Spain; jorgefrancisco.gonzalez@ulpgc.es

**Keywords:** fish parasites, Pintneriella, Trypanorhyncha, Cestoda, *Epinephelus marginatus*, dusky grouper, fish pathology

## Abstract

**Simple Summary:**

Trypanorhyncha are common parasites of marine fish. Despite numerous studies detailing their biology, knowledge on the effects caused by these parasites in fish tissues is still limited. Dusky groupers are keystone species, necessary for the preservation of several marine ecosystems. Considering their vulnerable state of conservation and the efforts being made to culture them, identification of the effects caused by Trypanorhyncha is vital. Here, we have assessed the prevalence of Trypanorhyncha in dusky groupers from the Canary Islands and the associated pathological changes. Of the 28 fish examined, 27 presented trypanorhynch larvae. Macroscopically, in the abdominal cavity, there were numerous larvae-filled cysts and nodules embedded in abundant fibrosis, hindering the separation of the organs. Microscopically, in the peritoneum, stomach and intestine, there were numerous degenerated parasitic cysts and extensive deposition of fibrous connective tissue with minimal inflammatory responses. This study shows that Trypanorhyncha are common parasites of adult dusky groupers from the Canary Islands. Even though the immune system appears to isolate and eliminate the parasites, extensive fibrosis may have a detrimental impact on fish health when adjacent organs are compressed and their functions impaired.

**Abstract:**

Trypanorhyncha are cestodes commonly infecting marine fish. Numerous studies have detailed the biology of Trypanorhyncha species, but information on the pathological changes produced by these parasites is limited. Dusky groupers are keystone species necessary for the preservation of several marine ecosystems. Considering their vulnerable state of conservation and the efforts being made to culture them, identification of the effects caused by Trypanorhyncha is vital. Here, we aimed to determine the prevalence and pathological changes produced by Trypanorhyncha in dusky groupers from the Canary Islands. The prevalence of trypanorhynch plerocerci was 96%. Grossly, in the abdominal cavity, there were numerous larvae-filled cysts and nodules. These were embedded in abundant fibrosis, producing visceral adhesions. Histologically, affecting the peritoneum, stomach, and intestine there were numerous degenerated encysted plerocerci and extensive deposition of mature connective tissue. These findings indicate that Trypanorhyncha is highly prevalent in adult dusky groupers from the Canary Islands, producing a progressive and chronic response. Furthermore, fish immune system appears to attempt to eliminate the parasites through fibrous encapsulation. Nonetheless, extensive fibrosis may have a detrimental impact on fish health when adjacent cells or tissues are compressed and their functions impaired.

## 1. Introduction

Cestodes of the order Trypanorhyncha have been found in marine fish all over the world [1,2,3,4,5,6,7,8,9]. They are characterized by a scolex with two or four bothria [10] and a tentacular apparatus comprised of four retractable tentacles [11]. Tentacles are armed with numerous hooks arranged in complex patterns to adapt to the attachment site in the final host [12]. With a complex life cycle [12], most Trypanorhyncha species require a definitive host (elasmobranch fish) and a first (small crustacean) and a second intermediate host (teleost fish or invertebrate) [8,13], although in some cases, only a single intermediate host is necessary [14]. Alternatively, in some species, paratenic hosts may harbor the plerocerci until a final host is available [10]. In this case, larger teleosts may serve as paratenic hosts to bridge gaps in the food chain between smaller teleosts and the elasmobranchs [10].

*Pintneriella* (Yamaguti, 1934) is a genus of Trypanorhyncha within the family Rhopalothylacidae [11]. Four species of *Pintneriella* have been identified, *P. musculicola,* *P. gymnorhynchoides, P. pagelli* and *P. maccallumi* [15]. The adult stage of *Pintneriella* spp. inhabits the gastrointestinal tract of the definitive host [16] from where it releases the free-swimming coracidium larva into the marine environment [10,17]. Once free in the environment, these are ingested by a first intermediate host and converted into procercoids [16]. In turn, when the procercoid is ingested by a second intermediate host, it penetrates through the gut wall and encysts in the viscera or musculature, maturing into a plerocercus [18].

Dusky groupers, *Epinephelus marginatus,* are large predatory fish and keystone species in the rocky bottom’s ecosystems, occurring at depths of up to 250 m [19]. Being a high-priced species [20] highly appreciated for the quality of their flesh [21], dusky groupers became a popular species among commercial and recreational fisheries [19]. As a result, their populations have suffered a major decline over the past decades [19] due to over-harvesting, habitat destruction and juvenile extraction [22]. With the aim of replenishing wild fish stocks, some efforts have been made to develop aquaculture for this species [23]. To attain efficient and sustainable fisheries management, aimed at conservation of endangered species, it is essential to expand the knowledge of the diseases affecting fish populations and their ecosystems [19]. 

Over the years, several studies have documented infections by Trypanorhyncha in *Epinephelus* spp. [3,4,7,9,24]. Despite considerable progresses in the morphological and taxonomical aspects of Trypanorhyncha species, information regarding the pathological changes caused by these parasites is still limited. A previous study documented the presence of *Pintneriella musculicola* in *Epinephelus* spp. from the Arabian Gulf [25]. The authors [25] detailed the presence of long, whitish plerocercoids of *P. musculicola* in the muscle, with associated muscle fiber atrophy and oedema. Inflammation, necrosis and fibrosis of the skeletal muscle, abdominal cavity, mesentery and liver have also been reported in Areolate groupers *Epinephelus aerolatus* infected with *Floriceps* sp. [26]. The presence and gross presentation of trypanorhynch larvae in *Epinephelus* spp. were briefly referred to in a few other studies [3,27] but further details were not provided. Interestingly, in another work reporting an infection by trypanorhynch larvae in *E. marginatus,* pathological changes were not observed [28]. 

In elasmobranch fish from the region, *P. gymnorhynchoides* was reported in Portuguese dogfish, *Centroscymnus Coelolepis,* from Azores [29], and *P. maccallumi* was found in smooth-hounds *Mustelus* spp. from the east Atlantic Ocean [15]. Given the presence of *Mustelus* spp. in the region, these may act as final hosts for *Pintneriella* sp. To the best of our knowledge, trypanorhynch infections have not yet been reported in *Epinephelus* spp. from the Macaronesia. 

Considering the large number and diversity of species of the order Trypanorhyncha, their high prevalence in a wide array of marine fish, the impact they might have on fisheries and the threat they may represent for mariculture species [30], knowledge on the pathological effects of Trypanorhyncha in fish is paramount. Based on this premise, the aim of the present study was to assess and determine the prevalence and pathological changes caused by trypanorhynch larvae in wild dusky groupers from the Canary Islands, Spain. 

## 2. Materials and Methods

### 2.1. Sample Collection

Between 2016 and 2018, a total of 28 specimens of adult dusky grouper were collected from the Eastern Central Atlantic from the Canary Islands. All fish were wild caught by professional fisherman and submitted dead to the Institute of Animal Health and Food Safety (IUSA), University of Las Palmas de Gran Canaria (ULPGC) for sanitary control within the Official Control Program of Ciguatera. 

**Figure 1:**
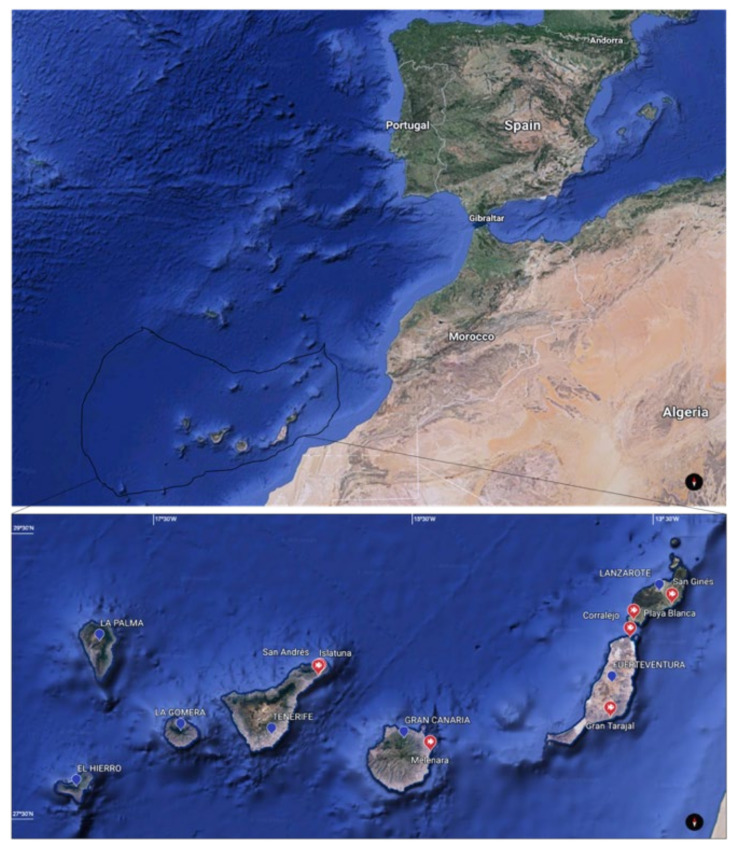
Spain established Exclusive Economic Zone in the Eastern Central Atlantic Ocean [31]. Inset: Capture zones in the Canary Islands (FAO fishing areas 34.1.2) [32] (maps obtained by Google Earth).

Though the exact catch location of the fish could not be determined, the date and approximate location of the capture were supplied. Dusky groupers were brought from Tenerife (*n* = 5), Gran Canaria (*n* = 1), Fuerteventura (*n* = 3) and Lanzarote (*n* = 19) (Figure 1). 

Total length (cm) and weight (kg) were measured for each specimen. Fish were necropsied, and samples of skeletal muscle, peritoneum, liver, spleen, stomach, intestine, gonads, swimbladder, kidney and heart were collected. Sex identification was determined by gross morphology of the gonads and confirmed by histology. 

### 2.2. Histology and Parasitology

Representative segments of the collected tissues were stored in 10% neutral buffered formalin for histopathological examination. Encapsulated larvae were detached from the tissues, and the capsule was removed under a stereoscope (Motic SMZ-171 TL). Larvae were washed with saline solution and fixed in both 10% buffered formalin and 70% ethanol. The formalin-fixed tissues were then embedded in paraffin, sectioned at 4 μm and stained with hematoxylin and eosin (HE) and Masson’s trichrome (MT) for histopathological assessment. Larvae samples stored in 70% ethanol were examined unstained under a stereoscope and an optical microscope (Olympus BX51TF). Stereoscope images (Moticam 1080) and photomicrographs of the larvae of the Trypanorhynch were taken (Olympus DP21).

## 3. Results

### 3.1. General Observations

Dusky groupers had an average body length and weight of 103.6 ± 6.2 cm and 22.4 ± 3.5 kg, respectively. Twenty-five were males, and three were females. 

### 3.2. Gross Findings

**Figure 2:**
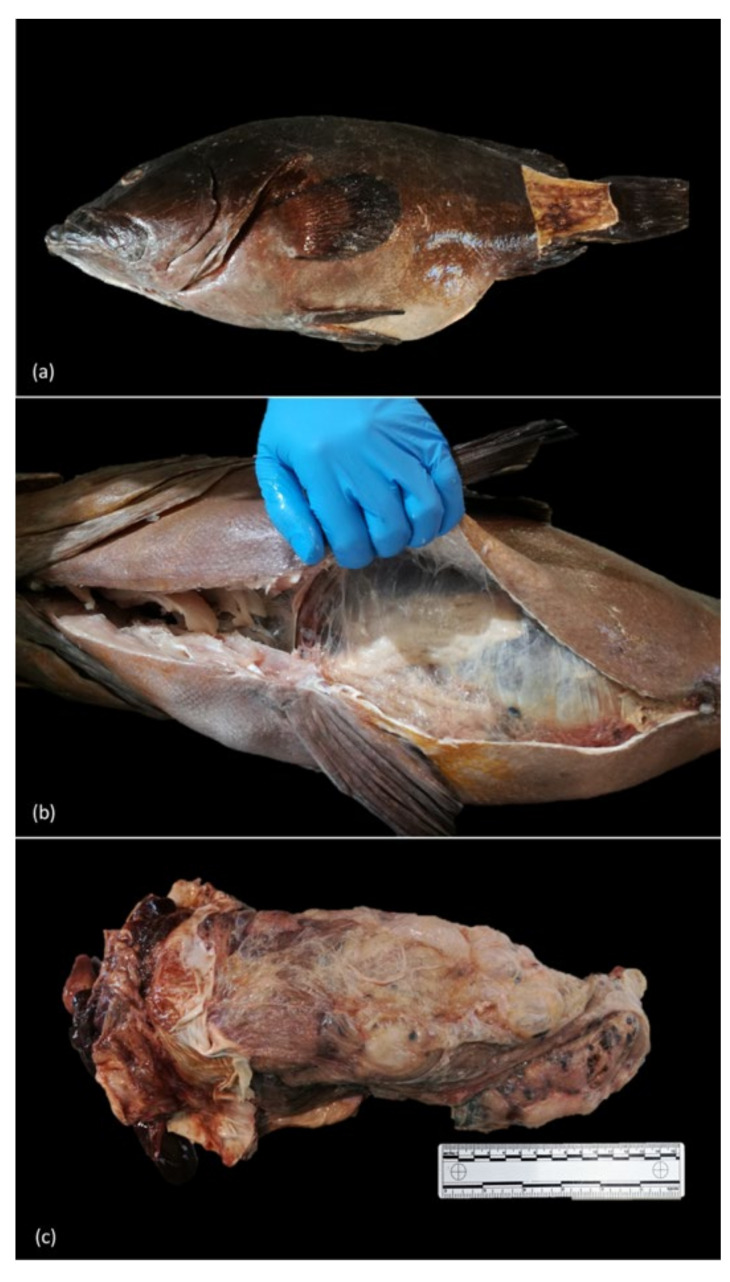
(**a**) Abdominal distension. (**b**) Abundant deposition of fibrous connective tissue in the abdominal cavity, (**c**) with diffuse intraperitoneal adhesions, hindering the separation of the individual organs, with numerous tan cysts and dark brown to black irregular nodules embedded.

**Figure 3:**
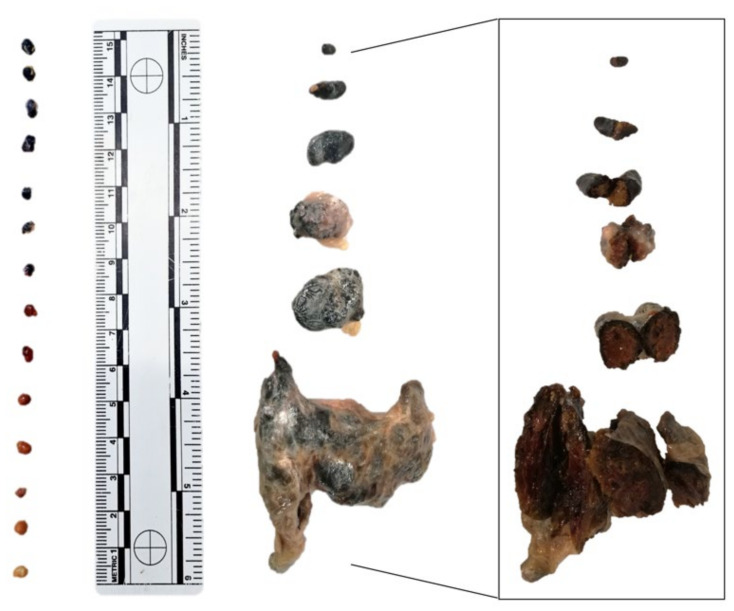
Size and color of the parasitic structures. Circular to oval, occasionally with a narrowed end, yellow to tan, larvae-filled cysts (**left**) and irregular, dark brown to black, firm to hard nodules (**center**) with a lighter gritty core at the cut surface (**right**).

External gross examination often showed distension of the abdominal cavity (Figure 2a). Internally, 27 out of 28 animals sampled presented numerous multifocal to coalescing parasitic cysts, attached or deeply embedded within the peritoneum and adhering to the serosal surfaces of the abdominal organs, often extending into the muscularis and submucosa of the stomach and intestine. These cysts were irregular, circular to oval with a narrowed end, variably sized (2 mm × 1.5 mm–6 mm × 3.2 mm), yellow to tan, larvae-filled, with small amounts of a serous fluid (Figure 3 left). Concurrently, there were numerous multifocal to coalescing dark nodules embedded within the fibrous tissue and adhering to the serosal surfaces of the celomic organs, occasionally extending into the muscularis and submucosa of the stomach and intestine and the hepatic parenchyma. These nodules were irregular, variably sized (10 mm × 25 mm–60 mm × 96 mm), dark brown to black, firm to hard, with a light brown and gritty core (Figure 3 right).

In the abdominal cavity, surrounding and adhering to the peritoneum, mesenteric fat and abdominal organs (i.e., stomach, intestine, liver, spleen, kidney, gonads), both of the above-mentioned structures were embedded in abundant bands of fibrous connective tissue with diffuse intraperitoneal adhesions, hindering the separation of the individual organs (Figure 2b,c). 

**Figure 4:**
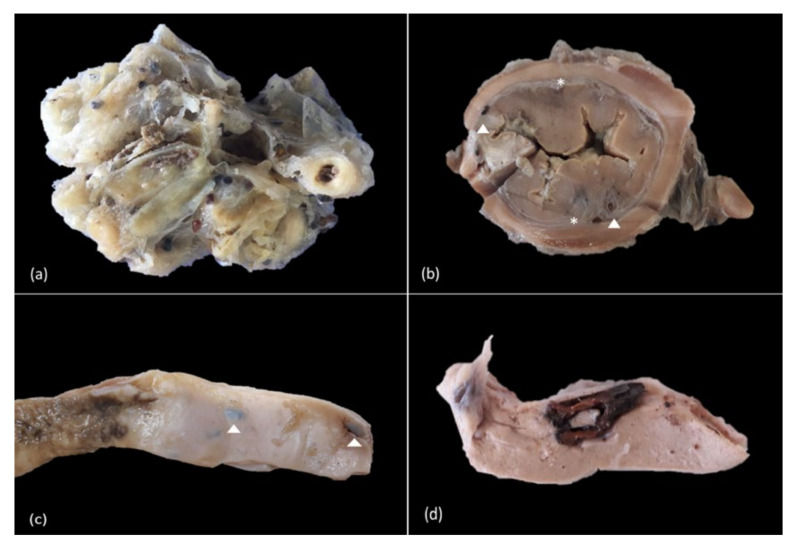
Location of the parasitic cysts and nodules. (**a**) Mesenteric fat and peritoneum, numerous cysts embedded in fibrous connective tissue. (**b**) Transversal section of the stomach, numerous cysts (arrowhead) in the submucosa with profuse deposition of mature fibrous connective tissue (*). (**c**) Intestine, multifocal, transmural larvae-filled cysts (arrowhead). (**d**) Liver, large dark brown nodule invading and displacing the hepatic parenchyma.

Larvae-filled cysts and nodules were mostly observed in the peritoneum and mesenteric fat, particularly surrounding the pyloric caeca (Figure 4a), and in the submucosa, muscularis and serosa of the stomach (Figure 4b) and intestine (Figure 4c). In a few cases, these structures were also present in the hepatic parenchyma (Figure 4d), adjacent to the gonads and adhering to the external wall of the bulbus arteriosus of the heart. 

Individualized parasites appeared as round to elongated, yellow to tan brown, soft, with an average size of 2 mm × 1.5 mm.

### 3.3. Histopathology

**Figure 5:**
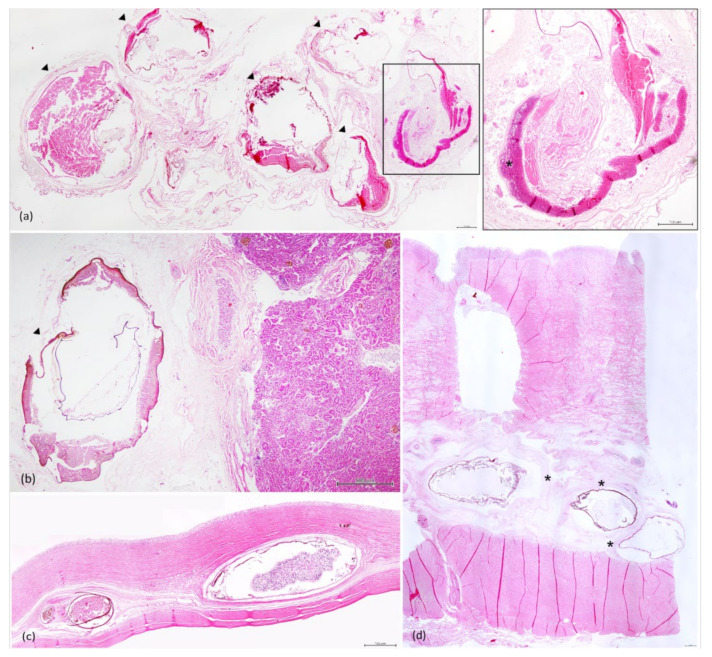
Histology, HE stain. (**a**) Peritoneum, cross-section of degenerated plerocerci (arrowheads) encapsulated in concentric layers of fibrous connective tissue (scale bar = 500 µm). Inset: severe inflammatory infiltrate and cellular debris (*) surrounding a ruptured cyst (scale bar = 500 µm). (**b**) Peritoneum, connective tissue adjacent to the kidney with a degenerated cyst (arrowhead) (scale bar = 500 µm). (**c**) Intestine, viable (**right**) and degenerated plerocerci (**left**) admixed with golden-brown macrophage aggregates (MA) (scale bar = 500 µm). (**d**) Stomach, numerous degenerated plerocerci embedded in a thick band of mature fibrous connective tissue (*) (scale bar = 500 µm).

Within the peritoneum (Figure 5a), adhered to the serosal surfaces (Figure 5b) and markedly expanding and compressing the muscularis and the submucosa of the stomach (Figure 5d) and intestine (Figure 5c), there were multifocal, variable-sized (up to 5.7 mm × 3.9 mm diameter), parasitic cysts containing a cross-section of a larval cestode (plerocercus). These were surrounded by an inner strongly basophilic, thin layer; a middle golden brown, thin cellular layer and an outer eosinophilic, thin layer of collagen with few fibroblasts. 

**Figure 6:**
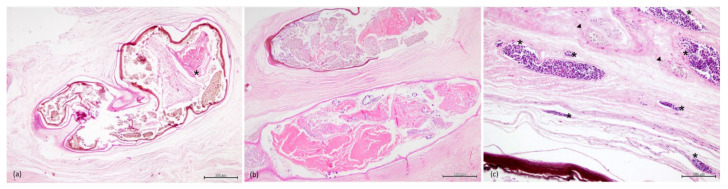
Histology, HE stain. (**a**,**b**) Encysted degenerated plerocerci, on occasions with fragments of the hooked tentacles (*), and finely stippled to coarse, strongly eosinophilic, golden-brown and basophilic debris (mineralization), enclosed by a fibrous capsule (scale bars = 500 µm). (**c**) Stomach submucosa, proliferation of small-caliber blood vessels (*) and MA surrounding (arrowheads) the degenerated plerocerci (scale bar = 100 µm).

Admixed and enclosed by a fibrous capsule, there were multifocally numerous, degenerated larvae, characterized by remnants of the plerocerci, on occasions with fragments of the hooked tentacles, and finely stippled to coarse, strongly eosinophilic, golden-brown and basophilic debris (mineralization) (Figure 6a,b). Surrounding the degenerated larvae cysts, there were often multifocal aggregates of golden-brown pigment-laden macrophages and, occasionally, scattered lymphocytes (Figure 6c). On occasions, in the peritoneum, when cysts were ruptured, there were variably sized aggregates of lymphocytes, and macrophages were observed surrounding and infiltrating the remnants of the parasite (Figure 5a).

**Figure 7:**
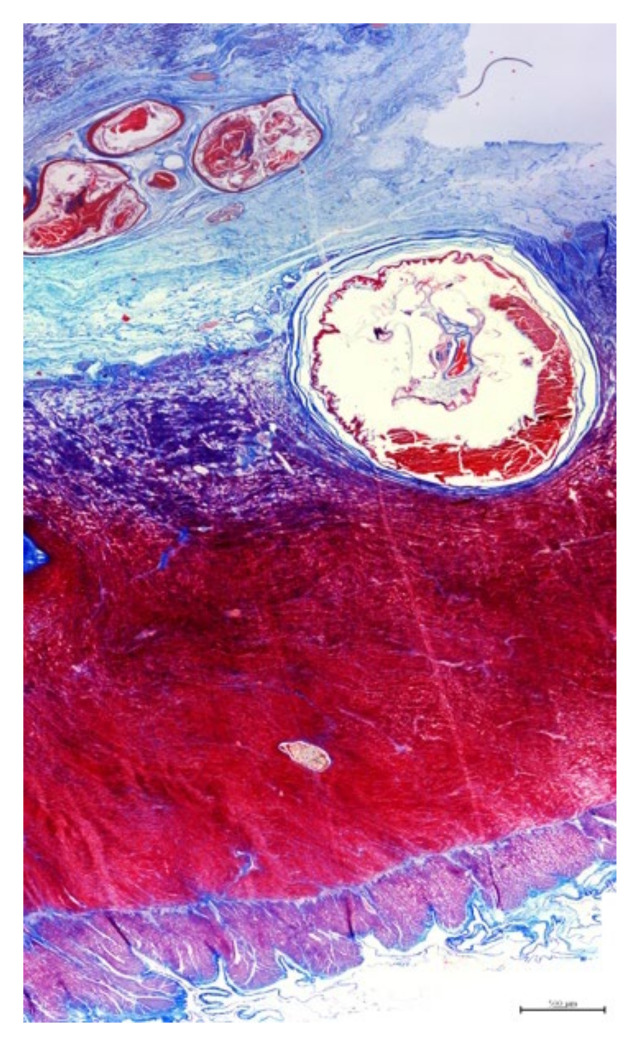
Histology, MT stain. Stomach, submucosa and muscularis adjacent to the cysts effaced and replaced by extensive areas of fibrous connective tissue (scale bar = 500 µm).

Surrounding the cysts, there was profuse deposition of mature fibrous connective tissue, characterized by densely packed collagen fibers, as highlighted by the MT stain (Figure 7), and minimal inflammation, with occasional interspersed small aggregates of lymphocytes and golden-brown macrophages and oedema. Diffusely, in the fibrotic areas of the submucosa of the stomach, there was proliferation of small-caliber blood vessels (Figure 6c). Furthermore, small and medium caliber arteries within the fibrous connective tissue had markedly thickened walls, expanded by dense layers of connective tissue with numerous small caliber blood vessels.

Occasionally, in the mucosa of the stomach, there were multifocal areas of mild necrosis and fibrosis.

The liver showed no significant pathological changes, but occasionally, hepatocytes were interspersed with small aggregates of lymphocytes and multifocal proliferation of macrophage aggregates (MA) with a golden-brown pigment. This proliferation of MA was also observed in the spleen, kidney, intestine and stomach.

**Figure 8:**
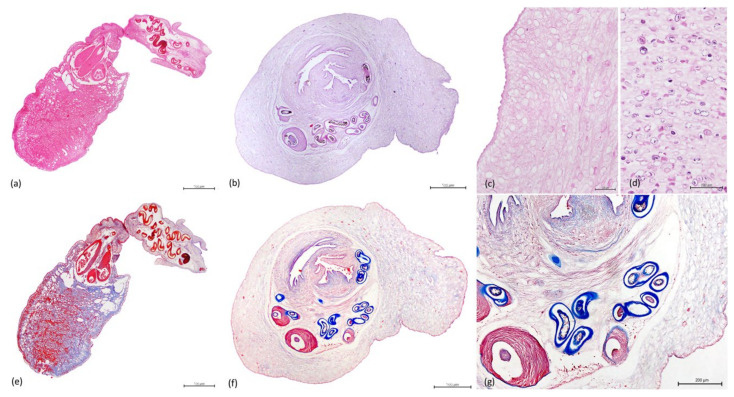
Histology, HE stain. Trypanorhyncha plerocerci. (**a**) Longitudinal section, eosinophilic tegument, lacy, fibrillar, eosinophilic parenchyma and scolex with muscular bulbs and armed tentacles (scale bar = 500 µm). (**b**) Transversal section, muscular bulbs and armed tentacles (scale bar = 500 µm). (**c**) Eosinophilic tegument (scale bar = 100 µm). (**d**) Parenchyma, numerous, basophilic to clear, round calcareous corpuscles (scale bar = 100 µm). Histology, MT stain. (**e**) Longitudinal section, tentacle sheaths and muscular bulbs staining red with MT (scale bar = 500 µm). (**f**) Transversal section, muscular bulbs and armed tentacles (scale bar = 500 µm). (**g**) Detail of the muscular bulbs and hooks (scale bar = 200 µm).

Sections of the individualized plerocerci showed an eosinophilic tegument and lacy, fibrillar, eosinophilic parenchyma with numerous, basophilic to clear, round calcareous corpuscles embedded and a scolex with four muscular bulbs and armed tentacles. Calcareous corpuscles are often dissolved during the fixation or histological processing, in which cases, the shape of the corpuscles remains, but they present as clear oval structures [33]. In some sections, the anterior end of the plerocerci was observed with the scolex, characterized by muscular fibers concentrically arranged (muscular bulbs) (Figure 8a–d), stained red by MT and the armed tentacles lined by series of refractile hooks (Figure 8e–g).

Severity and extension of the lesions was directly proportional to the intensity of the infection. Fish with a higher number of parasites (*n* > 30) presented extensive fibrosis of the peritoneum as well as the muscular and submucosa of the stomach and intestine. On the other hand, fish with a lower number of parasites (*n* < 30) showed less extensive fibrosis and the areas of the stomach and intestine affected were smaller. Differences were not observed between fish captured from different regions. However, the number of fish specimens received was not similar for all the islands; hence, it was not possible to establish any correlation. 

### 3.4. Parasitology

The overall prevalence of *Pintneriella* sp. larvae was 96.4% (27/28) with 3/3 specimens from Fuerteventura, 1/1 from Gran Canaria, 18/19 from Lanzarote and 5/5 from Tenerife islands. 

**Figure 9:**
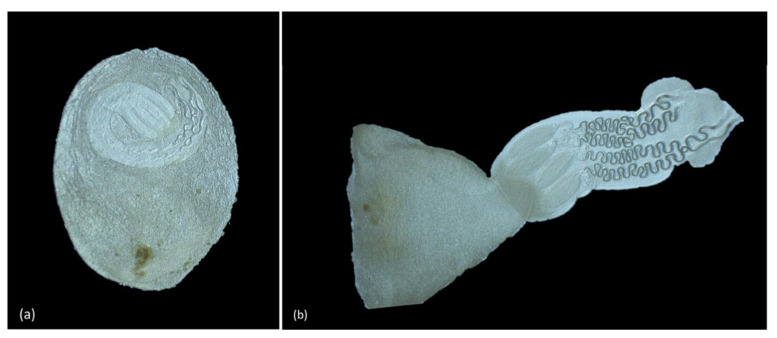
Plerocercus under stereoscope (0.75×). (**a**) Plerocercus within blastocyst. (**b**) Scolex of the plerocercus emerging from the blastocyst.

**Figure 10:**
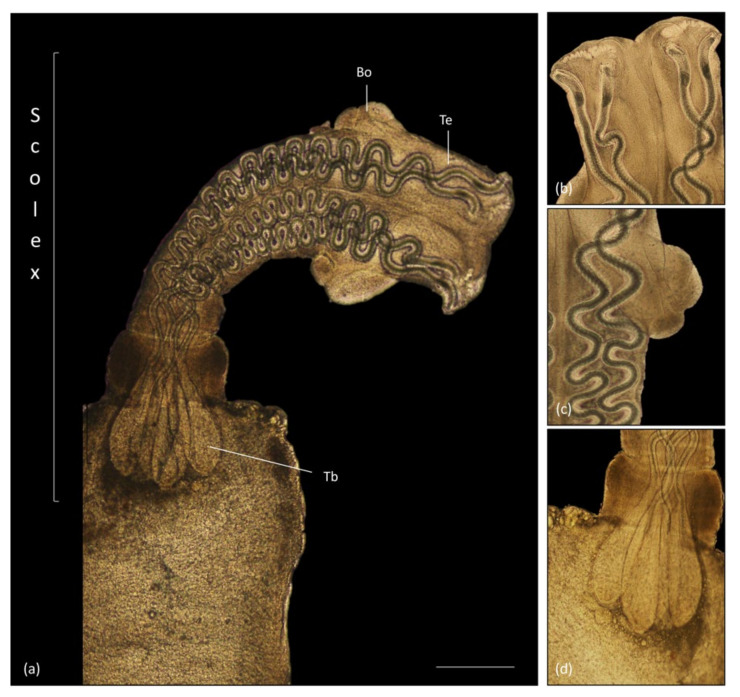
Plerocercus under a light microscope. (**a**) Scolex of the plerocercus emerging from the blastocyst (Bo—bothridium; Tb—tentacle bulb; Te—tentacle) (scale bar = 500 µm). (**b**) Detail of the hooked tentacles. (**c**) Detail of the bothridium. (**d**) Detail of the tentacle bulb.

Observation of the parasites preserved in 70% alcohol under a stereoscope and optic microscope showed two presentation forms of the plerocercus: a plerocercus enclosed within an oval, amber, blastocyst of 2 mm × 0.9 mm (Figure 9a) and a plerocercus with a blastocyst and a protruding scolex, amber, up to 10 mm × 2.1 mm (Figure 9b). The plerocercus showed a scolex peduncle (Figure 10a) with tentacles armed with hooks (Figure 10a,b), two bothridia (Figure 10b), pars vaginalis with the tentacle sheaths (Figure 10c) and pars bulbosa with the muscular bulbs (Figure 10d). Within the scolex peduncle, there was a characteristic rhyncheal apparatus with a metabasal and basal armature, tentacle sheaths and muscular bulbs. 

## 4. Discussion

Numerous studies have detailed the presence and morphological features of Trypanorhyncha species infecting groupers [3,4,7,9,24,25,26,28,34,35,36]. Notwithstanding, information on the pathological effects produced by these parasites remains limited. Therefore, the main goal of this study was to assess and determine the prevalence and pathological changes produced by Trypanorhyncha in dusky groupers caught in the Canary Islands, Eastern Central Atlantic Ocean. 

The prevalence of *Pintneriella* sp. in our study has been estimated as 96.4%. This result was considerably higher compared to those reported in previous studies. In *Epinephelus* spp. from the Arabian Gulf, the prevalence of *P. musculicola* and *Floriceps* sp. was around 15% [25] and 24.2% [26], respectively. Similarly, in white groupers, *Epinephelus aeneus,* and dusky groupers from the Turkish Mediterranean coast, the prevalence of *Grillotia* sp. was 17.7% and 18.4%, respectively [28]. In groupers from the Red Sea, the overall prevalence of *Callitetrarhynchus gracilis* was 9.2% [35]. Furthermore, in areolate groupers [37] and the orange-spotted grouper, *Epinephelus coioides* [36], from Bali, the prevalence ranged from 3.3% to 8.6%. These variations may be due to differences in the availability of the first intermediate host as prey [7,38], ontogenetic diet shifts [19,39,40] and the amount of prey usually ingested [41]. Larger fish typically feed on macrocrustaceans, smaller fishes and cephalopods [19,20] and need greater amounts of food, hence being more likely to ingest first intermediate hosts [41]. Fish from our study had a larger average length when compared to groupers from previous studies. Similarly, it has been observed in previous studies that trypanorhynch larvae occurred primarily in larger fish [25,26,42]. 

Grossly, fish presented with a distended abdominal cavity with diffuse and extensive fibrosis in the peritoneum, mesenteric fat and serosal surfaces of the abdominal organs. Embedded within this fibrous tissue, there were numerous, round with a narrowed end, yellow to tan-brown, larvae-filled cysts and irregular, dark brown to black, firm to hard nodules. These structures were also observed in the muscularis and submucosa of the stomach and intestine and, on a few occasions, invading the hepatic parenchyma and the pericardial cavity. In *Epinephelus* spp. from the Arabian Gulf [25], long, whitish plerocercoids of *P. musculicola* were observed in the muscle and were associated with muscle fiber atrophy and oedema [25]. Here, plerocercoids of *Pintneriella* sp. were not observed in the muscle, despite the similarities shared regarding the parasite genus and the host. In areolate groupers infected with *Floriceps* sp., Ibrahim [26] did not observe external lesions, but the liver showed atrophy with focal firm and necrotic areas. Fibrosis and adhesion of the abdominal organs were also observed [26]. These results differ from the findings presented here. In our study, the liver was only occasionally affected by dark brown nodules and scattered lymphocytes and macrophage aggregates, but atrophy was not observed. The presence and gross features of trypanorhynch larvae in *Epinephelus* spp. was also briefly mentioned in a few other studies. In dusky groupers from the Libyan coastal waters, larvae-filled cysts were mostly present in the head kidney, followed by the external surface of the stomach, ovary, and testis [27]. In our study, no evidence of plerocerci was detected in the head kidney. Plerocerci enclosed within both white and brown to black capsules were also recovered from the body cavity attached to the mesentery of *Epinephelus* spp. from Australia and New Caledonia [3]. Contrary to our results, pathological changes were not observed in dusky groupers from the Turkish Mediterranean coast infected with trypanorhynch plerocerci [28]. 

Histologically, we observed in the peritoneum and mesenteric fat, extensive areas of fibrosis with embedded plerocerci cysts adhering to the serosal surfaces of the abdominal organs and invading the muscularis and the submucosa of the stomach and intestine. Areas of the muscularis and submucosa adjacent to the cysts were often effaced and replaced by fibrous connective tissue with proliferation of macrophage aggregates with a golden-brown pigment and small caliber blood vessels. Occasionally, in the mucosa of the stomach, there were multifocal areas of mild necrosis and fibrosis. Similarly, fibrosis of the mesentery with adhesion of the internal organs and encapsulation of the plerocerci with fibrous connective tissue were also reported in areolate groupers infected with *Floriceps* sp. [26]. However, contrasting with our findings, those lesions were also accompanied by severe degenerative and necrotic changes in the liver and marked tissue destruction with intense inflammatory reaction in the skeletal muscle, caused by the entrance of the motile larvae [26]. By contrast, other studies reporting trypanorhynchs in fish tissues did not detail associated pathological changes [28], and some have suggested that these parasites were not likely to cause significant pathological changes [27,43]. 

Extensive proliferation of connective tissue with encapsulation of the plerocerci appears to be a common response in long-standing infections with cestodes [44,45,46,47,48]. Containment in this way serves to separate the parasite from the tissues to prevent further damage to the host [45,46]. Another reported trait in long-standing cestode infections is a marked decrease in the number of cells participating in tissue reaction and the predominance of fibrous connective tissue [44,49]. In most vertebrates, there is a particular immune response implicated in the production of collagen [50], a major component of mature connective tissue and an important agent of tissue repair [51] that is believed to play a role in both parasite encapsulation and tissue reconstruction [50]. Proliferation of small caliber blood vessels admixed with the connective tissue has been associated with an attempt to repair the injured tissues to get the necessary nutrients and oxygen [51]. In heavily infected fish, fibrosis tends to extend throughout the body cavity, causing adhesion of the viscera to each other and to the body wall [45]. When tissue injury is severe or recurring or if the wound-healing response is not appropriately regulated, it will result in overzealous or persistent wound-healing responses, becoming detrimental and contributing to the development of fibrotic pathology [50,52]. In fish, cestode infections have caused extensive fibrosis, producing compression and atrophy of the adjacent abdominal organs [53]. In severe cases, fibrosis may eventually lead to organ malfunction and death [52,54]. 

Macrophage aggregates (MA) are commonly found in the spleen, kidney, and liver in some fish species [55]. However, mobilization and proliferation of MA [55] may also be features of a chronic inflammatory response [55,56]. It has been shown that these pigment-laden aggregates may be found surrounding and within the encapsulating response of parasites [47,55,57], as observed in our study. Thus, MA are likely to act as collections of scavenging cells stimulated by excessive degenerating tissue [58]. 

Color variations grossly observed in the yellow to tan-brown larvae-filled cysts and dark brown to black nodules may be due to the deposition of different types of pigments. Histologically, in the larvae-filled cysts, the plerocerci remained intact. On the other hand, dark brown to black nodules represented the degenerated parasites. Necrotic remnants of the plerocercus admixed with finely stippled to coarse, strongly eosinophilic to golden-brown pigmented debris and basophilic pigment (mineralization) were lined by a layer of golden-brown cellular debris and further bounded by thick layers of mature connective tissue. Mineralization has often been described with degenerated cestodes [44,49,59]. It has been said to occur in older infections resulting in the death of the parasite [60]. Dystrophic calcification typically occurs in areas of necrosis when dead and dying cells may no longer regulate the influx of calcium into their cytosol, and calcium accumulates in the mitochondria. Calcium deposition is common with dead parasites. Their significance is that they are an indicator of previous injury to a tissue [61]. This, together with the proliferation of pigmented MA surrounding, and most likely within, the degenerated plerocerci were possibly the cause for the darker coloration observed in the dark brown to black nodules. In line with this, Beveridge et al. [3] remarked that brown and black envelopes contained only remnants of plerocerci and that this dark coloration was likely due to melanisation of the cyst wall. In some species of serranids, fibrotic encapsulation has been associated with what appears to be ceroid, lipofuscin, and melanin pigmentation [61], likely from the proliferation of MA. This seems to indicate that dusky groupers have an immune mechanism to contain, mineralize and eliminate these parasites. The high number of degenerated parasites seems to suggest that fish may develop immunity to these parasites, resulting in the death of the larvae, as previously proposed by MacKenzie [62]. In line with this, Rigby and Dufour [63] proposed that darker nodules were the result of a host-initiated immune response that isolated and killed the parasite [63]. Based on the numerous degenerated plerocerci encapsulated by thick layers of connective tissue, it has been suggested that plerocerci possibly remain in fish tissues for a long period [64]. Pathogenicity of trypanorhynch larvae appears to be highly associated with the intensity of the infection [10]. Here, we have observed that fish with a higher intensity of infection exhibited a more exuberant pathological response, with extensive fibrosis and visceral adhesion. Butterfish, *Peprilus triacanthus,* with heavy infections with trypanorhynch larvae in the skeletal muscle had weight loss when compared with those with lower burdens [10,65]. In lizardfish, *Saurida tumbil,* high levels of parasitism by *C. gracilis* have been associated with a high mortality rate at one phase of the fish life cycle [66].

Two possible sources of transmission are suggested here: ingestion of infected crustaceans with procercoids or ingestion of smaller fish with the immature plerocercus. In a study with adult whiting *Merlangius merlangus*, Özer et al. [43] postulated that infection with *Grillotia erinaceus* was likely to have occurred from the ingestion of smaller whiting carrying the first developmental stage. Likewise, in a study with halibut *Hippoglossus hippoglossus*, it was suggested that larger fish became infected by feeding on smaller fish containing recently acquired parasites not yet developed beyond the procercoid stage [67]. 

A crucial requirement for efficient fishery management is knowledge of the health and wellbeing of fish populations and their ecosystems [19]. Knowledge leads to better conservation and sustainable fishery management of the endangered species [19]. In this respect, assessment of the health condition of wild populations is vital, not just for wild stocks, but also for cultured fish that are also susceptible to infectious agents transmitted by broodstock that naturally live in or in the surroundings of the net cages [30]. Even though these infections do not appear to represent a threat for human health, recent research has shown that ingestion of fish with Trypanorhyncha can cause allergic disorders, since immunological hypersensitivity has been demonstrated in studies using murine models [68,69,70]. In addition, heavy cestode infections may reduce the fish market value by making them unappealing to consumers [8,16]. 

## 5. Conclusions

Our findings indicate that Trypanorhyncha are highly prevalent in adult dusky groupers from the Canary Islands. Infections by these parasites induce a progressive and chronic response characterized by an extensive and marked fibrotic reaction with encapsulation of the plerocerci. This suggests that the fish immune system attempts to eliminate the parasites through fibrous encapsulation. However, in fish with high-intensity infections, severe fibrosis with visceral adhesions is common. In these cases, compression and atrophy of the adjacent abdominal organs may occur, eventually leading to organ malfunction and death.
